# Disulfidptosis: a new target for central nervous system disease therapy

**DOI:** 10.3389/fnins.2025.1514253

**Published:** 2025-03-05

**Authors:** Jing Chang, Danhong Liu, Yuqi Xiao, Boyao Tan, Jun Deng, Zhigang Mei, Jun Liao

**Affiliations:** ^1^College of Medicine, Hunan University of Traditional Chinese Medicine, Changsha, China; ^2^Institute of Clinical Pharmacology of Chinese Materia Medica, Hunan Academy of Chinese Medicine, Changsha, China; ^3^Hunan Provincial Hospital of Integrated Traditional Chinese and Western Medicine (The Affiliated Hospital of Hunan Academy of Chinese Medicine), Changsha, China; ^4^Department of Neurology, Hunan Provincial Hospital of Integrated Traditional Chinese and Western Medicine, Changsha, China; ^5^Key Laboratory of Hunan Province for Integrated Traditional Chinese and Western Medicine on Prevention and Treatment of Cardio-Cerebral Diseases, Hunan University of Chinese Medicine, Changsha, China; ^6^Vascular Biology Laboratory, Medical College, Hunan University of Chinese Medicine, Changsha, China

**Keywords:** disulfidptosis, thiometabolism, thiol/disulfide, central nervous system diseases, therapy

## Abstract

Disulfidptosis is a pathologic process that occurs under conditions of NADPH deficiency and excess disulfide bonds in cells that express high levels of SLC7A11. This process is caused by glucose deprivation-induced disulfide stress and was first described by cancer researchers. Oxidative stress is a hypothesized mechanism underlying diseases of the central nervous system (CNS), and disulfide stress is a specific type of oxidative stress. Proteins linked to disulfidptosis and metabolic pathways involved in disulfidptosis are significantly associated with diseases of the CNS (neurodegenerative disease, neurogliomas and ischemic stroke). However, the specific mechanism responsible for this correlation remains unknown. This review provides a comprehensive overview of the current knowledge regarding the origin elements, genetic factors, and signaling proteins involved in the pathogenesis of disulfidptosis. It demonstrates that the disruption of thiometabolism and disulfide stress play critical roles in CNS diseases, which are associated with the potential role of disulfidptosis. We also summarize disulfidptosis-related drugs and highlight potential therapeutic strategies for treating CNS diseases. Additionally, this paper suggests a testable hypothesis that might be a promising target for treating CNS diseases.

## 1 Introduction

Cell death maintains balance in morphogenesis by clearing damaged or obsolete cells during a state of physical health or illness (Newton et al., 2024). Programmed cell death occurs during the development of normal neurons to establish a spatial and temporal framework. Additionally, various types of cell death, such as pyroptosis, apoptosis, ferroptosis, and necrosis, which involve abnormal signaling cascades and interrelationships, are involved in the pathological mechanism of neurological disorders (Moujalled et al., 2021). Recently, a novel form of programmed death called disulfidptosis was proposed, the mechanism of which is the focus of cancer research. Disulfidptosis cell death genes are potentially linked to various cancer types and may function as candidate genes for cancer diagnosis, prognosis, and therapeutic biomarkers (Liu and Tang, 2023). Copper death genes may be associated with various cancer types and could function as potential biomarkers for cancer diagnosis, prognosis, and treatment (Liu and Tang, 2022). Boyi Gan and colleagues defined disulfidptosis in 2023 as high SLC7A11 expression and NADPH depletion, resulting in the suppression of cystine/cysteine conversion under conditions of glucose starvation, which leads to disulfide bond accumulation, disulfide stress, and collapse of the cytoskeleton. Pyroptosis is an immunogenic programmed cell death that effectively activates tumor immunogenicity and reprograms the immunosuppressive microenvironment to improve cancer immunotherapy. However, an overexpression of SLC7A11 promotes the biosynthesis of glutathione to maintain redox balance and combat pyroptosis (Zhu et al., 2024b). Apoptosis occurs in development, tissue homeostasis, and immune function. Unlike disulfidptosis, apoptosis does not typically involve protein aggregation induced by oxidative stress or is centered around dysfunction of the actin network (Xiao et al., 2024). Ferroptosis is a novel form of programmed cell death characterized by iron-dependent oxidative damage, lipid peroxidation, and the accumulation of reactive oxygen species (Zuo et al., 2022). Disulfidptosis is a form of cell death caused by oxidative reductive imbalance resulting from amino acid metabolism and glucose metabolism disorders (Wang et al., 2024). In the physiological system, the thiol disulfide redox involves the reduction of disulfide to thiol and the oxidation of thiol to disulfide (Ghosh et al., 2023). Disulfidptosis is caused by the imbalance between thiols and disulfides, resulting in disulfide stress. In contrast, ferroptosis is caused by lipid peroxidation and excessive oxidative stress due to iron ion deposition. Some researchers speculate that disulfidptosis is a specific form of oxidative stress-induced cell death that corresponds to diseases of various systems in the humaccn body, such as the respiratory, digestive, urinary, and reproductive systems (Chen et al., 2024).

The prevalence of central nervous system diseases (CNSD), such as brain tumors, neurodegenerative diseases (Alzheimer’s disease, Parkinson’s disease, etc.), and ischemic stroke has increased significantly, severely affecting general health conditions and imposing significant financial and societal strain on individuals affected by these conditions (Zhang X. et al., 2021). The accumulation of high levels of reactive oxygen species, neurotoxic substances, and inflammatory cytokines forms the pathological mechanism underlying CNSD, which results in dysfunction of the nervous system and the development of therapeutic targets for neurological diseases (Patel, 2016). A study revealed that deleting ETHE1, a mitochondrial sulfur dioxygenase involved in sulfide catabolism in encephalopathy, leads to fatal sulfide toxicity; these findings suggest that most mammalian brains have a very limited ability to break down sulfide and that sulfide accumulation can cause brain damage (Tiranti et al., 2009). Previously, the processes and causes of the diverse pathological mechanisms underlying CNS disorders were elusive; Central nervous system (CNS)-related diseases exhibit a high mortality rate and pose significant risks to both physical and mental health, making them a critical focus of research (He et al., 2024). Therefore, by conducting a literature review, we propose that thiometabolism is closely related to specific cellular redox reactions that depend on the thiol/disulfide ratio. A detailed mapping of disulfidptosis in nervous system disease may reveal the network regulating the crucial targets of the pathological process and provide new insights into potential clinical treatment directions for neurological disorders.

## 2 Research progress on factors related to disulfidptosis in the CNS

### 2.1 Physiological function of sulfur

**Figure 1:**
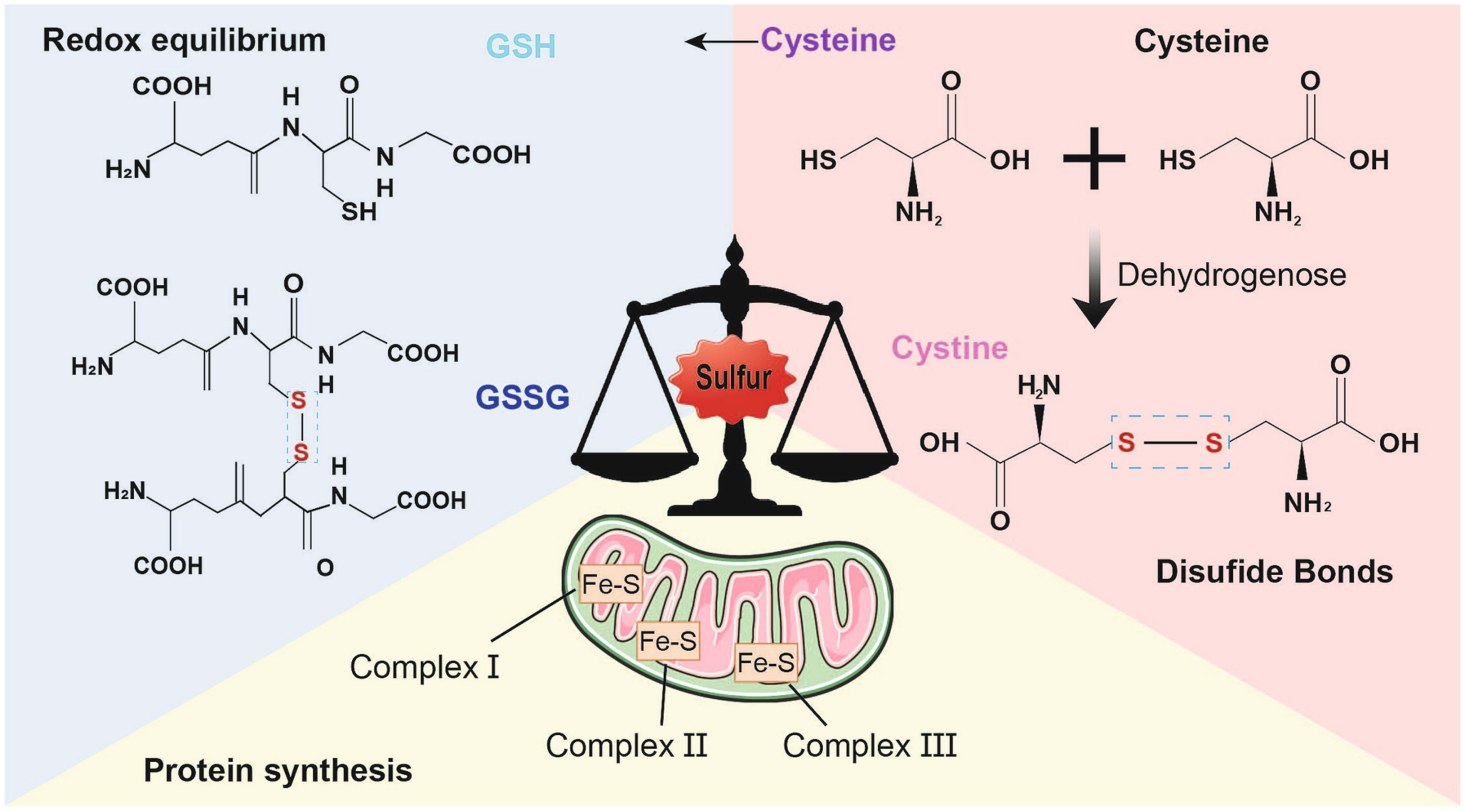
Physiological function of sulfur. Sulfur mainly exists in the form of compounds in the human body and participates in various biological processes. First of all, sulfur also participates in antioxidant defense and detoxification processes. Cysteine is an important antioxidant that can neutralize reactive oxygen species, thereby reducing the damage of oxidative stress to cells iron–sulfur proteins are minimally functional proteins of mitochondria; Secondly, Cystine and cysteine, two sulfur-containing amino acids, participate in the formation of disulfide bonds in proteins, stabilizing their three-dimensional structure and function. The formation of disulfide bonds can maintain the normal conformation of proteins, thereby ensuring their normal function; Finally, sulfur also participates in the synthesis of iron sulfur proteins and is an essential component of the electron transport chain. It plays a critical role in biological processes such as cellular respiration and photosynthesis.

Redox (oxidation–reduction) reaction is the core of the existence of life. The reactants namely, oxygen, nitrogen, and sulfur, mediate the redox control of a series of important cellular processes (Sies et al., 2024). Sulfur is present mainly in the form of compounds in the human body and is involved in various biological processes, including protein synthesis, energy metabolism, and oxidation–reduction equilibrium (Fan et al., 2023). First, sulfur is involved in the synthesis of sulfur-containing amino acids, such as cystine, cysteine, and methionine, as well as sulfuric acid, which play a vital role in sustaining normal physiological functions (Chatterjee and Hausinger, 2022). The mechanism by which cells maintain redox homeostasis or function in the antioxidant defense system strongly depends on the regulatory reactivity of the sulfur atoms inside or derived from cysteine and methionine (Miller and Schmidt, 2020). The antioxidant capacity of sulfur-containing amino acids, such as cysteine, contributes to their ability to neutralize reactive oxygen species (ROS) (Stipanuk, 2020). Additionally, two molecules of cysteine are converted into cystine through the action of dehydrogenase in the neuronal redox reaction, in which sulfhydryl, a functional group of thiols in cysteine, leads to the formation of disulfide bonds (Stipanuk, 2020). Proteins containing thiol-disulfide bonds play crucial roles in regulating cellular redox homeostasis and serve as diagnostic markers for diseases influenced by redox conditions (Hanchapola et al., 2023). The formation of protein disulfide bonds serves as an indicator of oxidative stress linked to neurodegeneration (Landino et al., 2014). Second, sulfur atoms are closely associated with the synthesis of iron–sulfur proteins, which are essential and minimally functional proteins of mitochondria; abnormal iron–sulfur clusters lead to deficiencies in target proteins, including complexes I, II, and III; aconitase; and lipoic acid (Selvanathan and Parayil, 2022). Third, in cellular metabolism, sulfur helps maintain redox balance, which results in the formation of a cellular antioxidant system that mediates intercellular and intracellular signaling (Moreno et al., 2014). Some studies have suggested that disulfide stress may be a distinct form of oxidative stress in cases of acute inflammation involving protein cysteine and gamma-glutamylcysteine, as well as cysteine/cystine oxidation. However, no alterations in glutathione (GSH) oxidation or protein GSH were observed (Ward and DeNicola, 2019). During cellular reduction and oxidation, the transformation of sulfur-containing proteins and the dynamic balance of thiol-disulfide bonds are associated with antioxidant defense in the development of several psychiatric disorders (Messens and Collet, 2013; Ergin et al., 2023) (Figure 1).

### 2.2 Elements of disulfidptosis in the CNS

The elements of disulfidptosis in the CNS involve the following factors: the Xc-system, the cystine/cysteine balance, glucose starvation, and NADPH depletion.

#### 2.2.1 Cystine/glutamate transporter (Xc-system)

The cystine/glutamate transporter (Xc-system) comprises disulfide-bonded heterodimers solute carrier family 7 member 11 (SLC7A11, also known as xCT) and SLC3A2, which form a membrane transport protein system that is responsible for transporting extracellular cystine and intracellular glutamate across the cell membrane at a 1:1 ratio; this process plays a crucial role in signaling transmission during antioxidant defense in the CNS (Bridges et al., 2012). The sudden hypoxia of neurons during a stroke results in a significant release of glutamate, causing hypoxic depolarization and subsequent rapid cell death. The latency of glutamate-driven Alzheimer’s disease events significantly influences the degree of subsequent tissue damage (Verbruggen et al., 2022; Heit et al., 2023). These findings indicate that glutamate self-stabilization and balance of oxidative stress are important tasks for systemic Xc-completion in the nervous system (Dahlmanns et al., 2023). Glutamate is the primary neurotransmitter that stimulates neurons in the central nervous system (CNS), where it initially begins fast signaling at the synapse and is subsequently reabsorbed by peripheral glial cells. For example, transporters 1 (EAAT1) and 2 (EAAT2), which take up synaptic glutamate to maintain its normal extracellular levels found in astrocytes, prevent glutamate accumulation in the synaptic gap and in the nervous system. Upregulation of SLC7A11 increases glutamate output in cells, whereas downregulation of EAAT reduces intracellular glutamate input (Dahlmanns et al., 2023). On the other hand, the function of xCT is to introduce cysteine for glutathione biosynthesis and antioxidant defense (Wang et al., 2023). Under glucose starvation conditions, damage to the pentose phosphate pathway can lead to a decrease in its metabolic product NADPH, resulting in a reduction in NADPH electron donors that prevent cystine from being converted to cysteine. Therefore, overexpression of the Xc system can induce inappropriate accumulation of cystine in the cytoplasm, leading to disulfide stress (Zhong et al., 2023). These findings confirm the theory that Xc-system serves as a link between inflammation and glutamate excitotoxicity and that xCT might act as a target for reducing glutamate excitotoxicity in neurodegenerative diseases under inflammation (Pampliega et al., 2011). Consequently, abnormal mechanisms of cystine and glutamate exchange make the Xc-system a potential contributor to many CNSD (Adla et al., 2024).

#### 2.2.2 Cystine/cysteine balance

Cystine and cysteine contain disulfide bonds and thiols, respectively, and can undergo interconversion. Cysteine is an integral part of the main antioxidant GSH and acts as a potent antioxidant in the brain, playing a crucial role in protein synthesis and redox homeostasis (Paul et al., 2018). The conversion of cystine to cysteine is required to maintain the thiol/disulfide redox equilibrium within cells (Go and Jones, 2005). Cystine and other disulfide compounds accumulate in large quantities under conditions of high SLC7A11 expression, glucose deprivation, and NADPH depletion, resulting in disulfide stress (Liu X. et al., 2024). Dynamic regulation of thiol/disulfide homeostasis is essential for various metabolic processes, including signal mechanisms, inflammation, and antioxidant defense (Erenler and Yardan, 2017). Thiol/disulfide is a critical component of the antioxidant defense system and is necessary for maintaining the intracellular redox balance and for the diagnosis and prognostic assessment of potentially lethal diseases (Chatterjee and Hausinger, 2022). Restoring cysteine homeostasis has therapeutic benefits in neurodegenerative diseases (Paul et al., 2018). Therefore, the level of cystine/cysteine, which can be used to detect thiol/disulfide, is an important indicator of disulfide stress.

#### 2.2.3 Glucose starvation and NADPH depletion

Glucose is the main source of NADPH production through the pentose phosphate pathway (PPP) (Ying et al., 2021). Nicotinamide adenine dinucleotide phosphate (NADPH) is synthesized by four enzymes in mammalian cells, including isocitrate dehydrogenase, malic enzyme glucose-6-phosphate dehydrogenase (G6PD), and PGD; the oxidized form is NADP+. Glucose-6-phosphate dehydrogenase (G6PD), which serves as a primary source of NADPH oxidase, a reducing agent, and a hydrogen ion donor in the reduction reaction, plays multiple roles in energy supply, signaling, and antioxidant reactions (Stanton, 2012; Zhang and Peng, 2014). NADPH, as a coenzyme, can act as a reducing agent for the conversion of cystine to cysteine, playing a role in the transfer of hydrogen in the reduction reaction (Mi et al., 2024). Glucose starvation damages the glycolysis and pentose phosphate pathways, leading to an increase in reactive oxygen species (ROS) production and damage to the antioxidant system, resulting in oxidative stress, redox imbalance, and cell death (Ren and Shen, 2019). When glucose starvation occurs, a large amount of NADPH is consumed, and the NADP+/NADPH ratio significantly increases. Cystine in SLC7A11-overexpressing cells cannot be reduced to cysteine, resulting in increased levels of disulfide and disulfidptosis (Liu et al., 2023a). For a long time, glucose has been an indispensable fuel for the brain, as it can perform many key functions, including producing ATP, managing oxidative stress, and synthesizing neurotransmitters, neuromodulators, and structural components (Dienel, 2019). Dysfunction of glucose metabolism in the entire brain or specific cell types, including ischemic brain injury and neurodegenerative diseases, is closely related to neurological pathology (Zhang S. et al., 2021). Under neuropathological conditions, mitochondrial defects often lead to electron transfer processes and reduced NADPH oxidase activity, resulting in increased reactive oxygen species production, which weakens the redox buffering capacity of the cell and may damage key enzymes involved in energy metabolism (Tang, 2020).

Disulfidptosis is associated with the equilibrium of some redox regulatory pairs, such as cystine vs. cysteine and NADP+ vs. NADPH (Wang et al., 2024). During disulfide reduction, NADPH plays a crucial role by transferring electrons and serves as a precursor for synthesizing enzymes for TRX-disulfide reductase (TrxRs) and glutathione-disulfide reductase (Gsrs) (Miller et al., 2018). NADPH participates in the generation of reducing antioxidants, such as GSH and thioredoxin (Trx), to prevent redox stress. According to some researchers, NADPH is a double-edged sword in redox reactions; although it inhibits oxidative stress in the cellular antioxidant system, it serves as a substrate for ROS production by NADPH oxidases (NOx), exacerbating oxidative damage (Liu et al., 2021). The cystine/cysteine system strongly regulates the mechanism of disulfidptosis, where the intracellular transport of cysteine is regulated by the Xc- system, and the conversion of cystine and cysteine is mediated by NADPH electron delivery. Additionally, disulfidptosis requires high consumption or a low supply of NADPH, high expression of SLC7A11, and glucose starvation.

### 2.3 Disulfide stress in disulfidptosis

Disulfide bonds play important roles in maintaining the rich characteristics of protein structure, stability, and function (Robinson and Bulleid, 2020). Disulfidptosis is a regulatory form of cell death induced by disulfide stress (Liu et al., 2023a), which is caused by an imbalance of the intracellular glutathione and thioredoxin antioxidant systems, leading to the accumulation of disulfide bonds.

#### 2.3.1 Disulfide stress effector protein: F-actin

The cytoskeleton is a three-dimensional structural network composed of interwoven protein fibers, primarily consisting of microtubules, microfilaments, and intermediate fibers. It maintains the unique shape of cells and is associated with cell movement (Schmid et al., 2024; Haseena et al., 2024). F-actin is an important component of microfilaments (MFs), which are spiral fibers composed of actin polymers that are ubiquitous in eukaryotic cells. When the actin monomer G-actin binds to ATP, the monomer is assembled into the polymer F-actin. When ATP is hydrolyzed to ADP, F-actin is depolymerized (Dominguez and Holmes, 2011). The proper function of actin strongly depends on its oxidation–reduction state; under oxidative stress, actin can become oxidized and undergo alterations in its shape and function (Rouyère et al., 2022). The cysteine residue in actin functions as a sensor for oxidative stress, resulting in a high level of sensitivity to ROS, RNS, and lipid peroxidation (Farah and Amberg, 2007). Under *in vitro* conditions, Cys 374 of actin has the highest reactivity, which leads to the formation of intramolecular disulfide bonds with Cys 285 or other actin molecules (Farah et al., 2011). F-actin plays a vital role in dendritic spines, maintaining synaptic structure and function, whereas disulfide stress may cause the collapse of the F-actin cytoskeleton, which is the pathological outcome of disulfidptosis (Li et al., 2024). In AD patients, the actin cytoskeleton is lost from synapses. Glutamatergic receptor numbers, neurotransmission, and synaptic strength are all affected when the actin cytoskeleton is lost, compromising synaptic integrity (Haseena et al., 2024).

#### 2.3.2 Disulfide stress-related Rac1-WRC-Arp2/3 signaling pathway

Rac1 (a type of Rho GTPase), a fundamental regulatory factor of the actin cytoskeleton, plays important roles in cell movement, polarity, and migration (Bailly et al., 2024). Furthermore, Rac1 plays a crucial role in specific brain functions, including neuronal migration, synaptic plasticity, and memory formation, through its regulation of actin dynamics in neurons. Abnormal expression and activity of Rac1 have been observed in various neurological disorders (Wang X. et al., 2020). Waves exist in pentamer complexes known as WAVE regulatory complexes (WRCs), including ABIs, NAP1 (also known as NCKAP1), CYFIPs, and HSPC300 (Alekhina et al., 2017). Preliminary CRISPR screening and functional studies revealed that inactivation of the WRC can promote actin polymerization, regulate actin cytoskeletal dynamics, and inhibit disulfidptosis (Liu et al., 2023a). WRCs also play crucial roles in regulating actin cytoskeletal dynamics and remodeling eukaryotic cells, including the regulation of dendritic spine growth and presynaptic assembly, which are linked to various brain diseases (Ibarra et al., 2005; Han and Ko, 2023). The removal of NCKAP1 and other WRC proteins weakens disulfide stress, whereas the excessive expression of constitutively activated Rac stimulates disulfidptosis in a WRC-dependent manner (Liu et al., 2023a). The NCKAP1 gene may regulate the expression of actin by modulating intracellular disulfide levels and the stability of the actin cytoskeleton. Rac1 can bind to WRC, inducing a conformational change that promotes the activity of the Arp2/3 complex, leading to F-actin nucleation and lamellipodia formation. This, in turn, facilitates actin polymerization and the formation of lamellipodia (Begemann et al., 2021). Consequently, the Rac1-WRC pathway facilitates the formation of disulfide bonds, thereby mediating actin polymerization in disulfide stress (Li et al., 2024).

#### 2.3.3 Disulfide stress-related disulfide bond regulatory system: the Trx and GSH/Grx systems

Thioredoxin (Trx) and the GSH/Grx system play important roles in maintaining redox balance in the brain, which is a tissue prone to oxidative stress due to its high energy demands. These two disulfide reductase systems are active in various regions of the brain and are considered key antioxidant systems in the central nervous system (Ren et al., 2017). The pathological mechanism underlying disulfidptosis is associated with a redox imbalance state, which involves the formation of disulfide bonds by sulfur oxidase and sulfate lyase, ultimately leading to disulfide stress (Wang et al., 2023). To suppress disulfidptosis, two protein repair systems, the Trx system and the glutaredoxin (Grx) system, balance the conversion of thiol groups and disulfide bonds. Both systems are essential for defending against oxidative damage through their disulfide reductase activity, which regulates the dithiol/disulfide balance (Shahriari-Farfani et al., 2019).

##### 2.3.3.1 Trx system

The Trx system is composed of Trx, thioredoxin reductase (TrxR), and NADPH (Bjørklund et al., 2022). Chloroplastic thioredoxins (Trxs), a family of thiol-disulfide oxidoreductases, are the products of two mammalian genes, txn1 and txn2, which encode the cytoplasmic and mitochondrial Trx isoforms, respectively (Kang et al., 2019). Trx regulates redox equilibrium in mammalian cells and can be triggered by multiple factors, including oxidative stress, inflammation, aging, and autoimmune disorders (Yang et al., 2024) Equations [1–3]. The Trx system reduces cystine accumulation by regulating cystine/cysteine balance, thereby preventing disulfidptosis (Kang et al., 2019). Trx catalyzes the thiol-disulfide exchange reaction, which involves electron transfer between Trx and its target protein. In subsequent programs, NADPH, as an electron donor and a mixed disulfide bond (Figure 3) is reduced by TrxR, and the reaction cycle can be represented as follows (Yang et al., 2024; Li et al., 2019; Zheng Z. et al., 2018).


(1) $$Trx-\left(SH\right)2+\text{Protein}-S2⇌\text{Trx}–S2+\text{Protein}–(\text{SH})2$$

(2) $$\text{TrxR}-S2+\text{NADPH}+H^{+}⇌\text{TrxR}–(\text{SH})2+{\text{NADP}}^{+}$$

(3) $$Trx-S2+\text{TrxR}-\left(SH\right)2⇌\text{TrxR}–S2+\text{Trx}–(\text{SH})2$$

##### 2.3.3.2 The GSH/Grx system

The GSH/Grx system plays key roles in controlling signaling and imbalance in thiol-disulfide redox homeostasis and redox reactions, which are linked to the development and progression of oxidative stress-related disorders (Chai and Mieyal, 2023), and the GSH-Grx system consists of NADPH, glutathione reductase, GSH and Grx (Lu and Holmgren, 2014). GSH-Grx belongs to the Trx protein family and facilitates the reduction of disulfide bonds to maintain redox homeostasis. There are two main forms of Grx: Grx1 is found in the cytoplasm and accepts electrons from GSH, whereas Grx2 is located in the mitochondria and nucleus of mammalian cells and can obtain electrons from GSH and TrxR2 (Fernandes and Holmgren, 2004). Like thioreductase, GSH can act as a dithiol reducing agent^[7CC4]^. Glutathione (GSH) is an important, naturally occurring small-molecule thiol composed of glutamate, cysteine, and glycine. GSH has antioxidant and detoxifying activities (Liu X. et al., 2020) and may also participate in physiological processes such as protein folding, thiol maintenance from oxidation, and cell cycle regulation (Averill-Bates, 2023). GSH is an important mechanism for maintaining brain redox balance and regulating several proteins that are crucial for neurobiological processes (Ogata et al., 2021). GSH is present in high concentrations in the brain (about 1–3 mM). Studies have shown that the Trx and GSH/Grx systems are specific to different organelles in neurons and glial cells and regulate redox signaling and that thiol-disulfide bond conversion (GSH) occurs in two states: oxidized and reduced (Horibe et al., 2001). The reduced form of GSH is generated in a reaction catalyzed by glutathione synthetase (GSS) and is transformed into glutathione disulfide (GSSG) through the oxidation of sulfhydryl residues. GSSG is then reduced back to GSH through interaction with glutathione reductase (the normal ratio of GSH/GSSG is about 100:1), using NAPDH as a cofactor (Couto et al., 2016; Georgiou-Siafis and Tsiftsoglou, 2023). Therefore, the GSSG-to-GSH ratio is a measure of the cellular redox status (Moujalled et al., 2021). Studies have shown that the redox couples cysteine/cystine (Cys/CySS) and Trx (SH)2 [reduced Trx/TrxSS] [oxidized Trx], as well as GSH [reduced glutathione] and GSSG [oxidized glutathione] are critical components of the thiol/disulfide redox system (Circu and Aw, 2011) (Figure 3). Oxidative stress caused by GSH deficiency is common in many CNS diseases (Adla et al., 2024), and supplementation with n-acetylcysteine (NAC) can prevent GSH deficiency as an independent treatment (Ruffmann and Wendel, 1991). Under oxidative stress, cysteine residues (-SH) and GSH on proteins create a reversible post-translational alteration called S-glutathionylation (Protein-SSG) (Dominko and Đikić, 2018). Grx is an important component of the thiol-disulfide oxidoreductase family, which catalyzes redox reactions between GSH and GSSG (Fernandes and Holmgren, 2004; Mustafa et al., 2021). Cysteine-179 of the β subunit of the inhibitory κB kinase (IKK) signaling complex is a core target of S-glutathionylation. Glutathione reductase (Grx) reverses the S-glutathionylation of IKK-β Cys179, thus restoring kinase activity (Reynaert et al., 2006).

**Figure 2:**
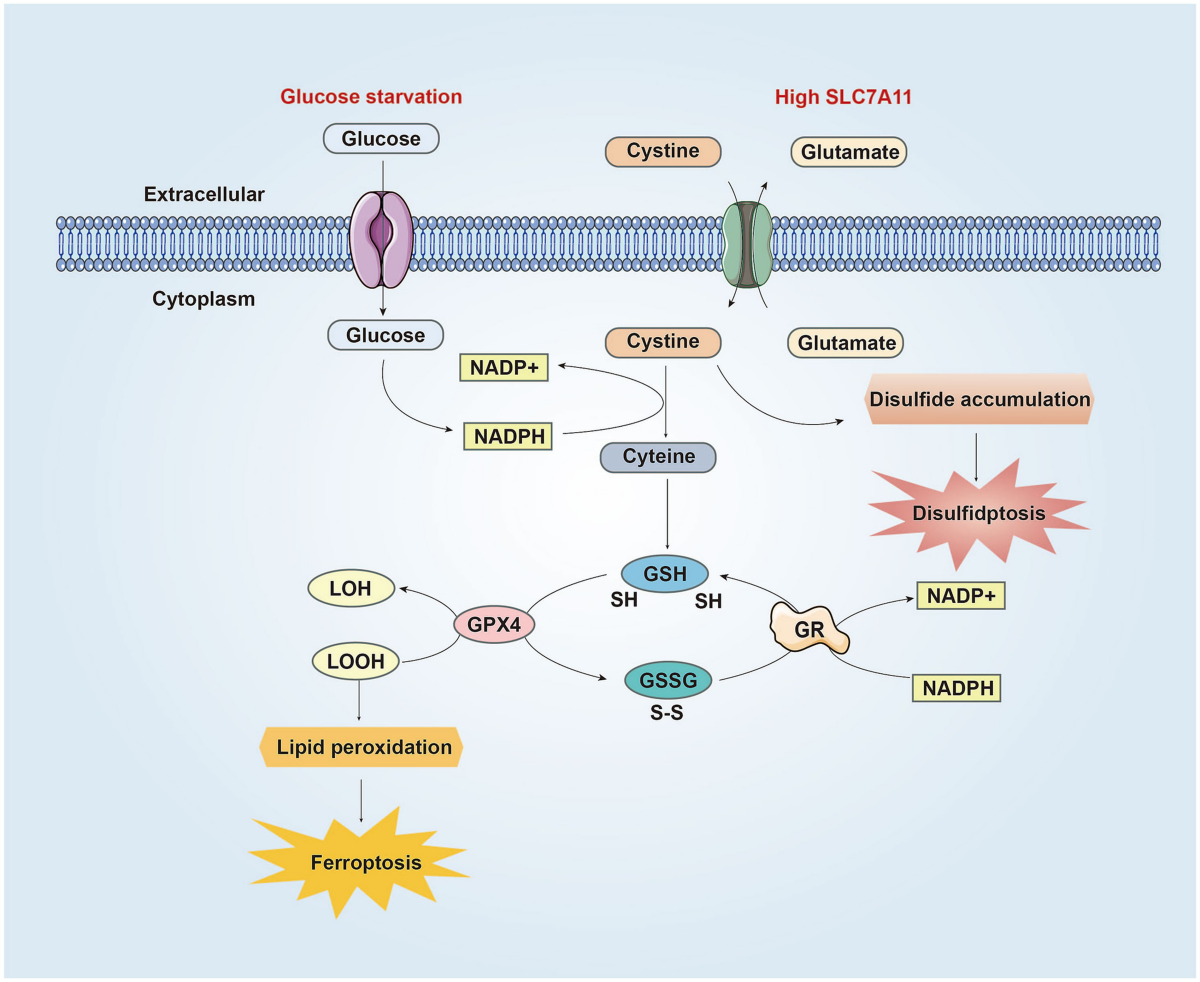
The similarities and differences between disulfidptosis and ferroptosis in CNS. SLC7A11 is the catalytic subunit of the XcT system, which absorbs cysteine from the extracellular environment and converts it into cysteine to synthesize GSH. GPX4 uses GSH to reduce LOOH to LOH, preventing lipid peroxidation and inhibiting ferroptosis. Meanwhile, GSH is oxidized to GSSG. Then GSSG is converted back to GSH through GR mediated reduction reaction, consuming NADPH in the process. Under conditions of glucose starvation and high SLC7A11, the pentose phosphate pathway is blocked, resulting in reduced NADPH production, hindered conversion of cysteine to cysteine, accumulation of cysteine and other disulfides, triggering the formation of abnormal disulfide bonds in redox sensitive proteins, ultimately leading to the rupture of the cytoskeleton and cell disulfidptosis. On the other hand, due to NADPH depletion, cystine cannot be converted into cysteine, resulting in reduced synthesis of GSH and generation of lipid peroxides, leading to ferroptosis. Abbreviations: NADP+: nicotinamide adenine dinucleotide phosphate, reduced form; NADPH: nicotinamide adenine dinucleotide phosphate; GSH: glutathione; GPX4: Glutathione peroxidase 4; LOOH: lipid hydroperoxides; LOH: lipid alcohols; GSSG: glutathione disulfide; GR: glutathione disulfide reductase.

**Figure 3:**
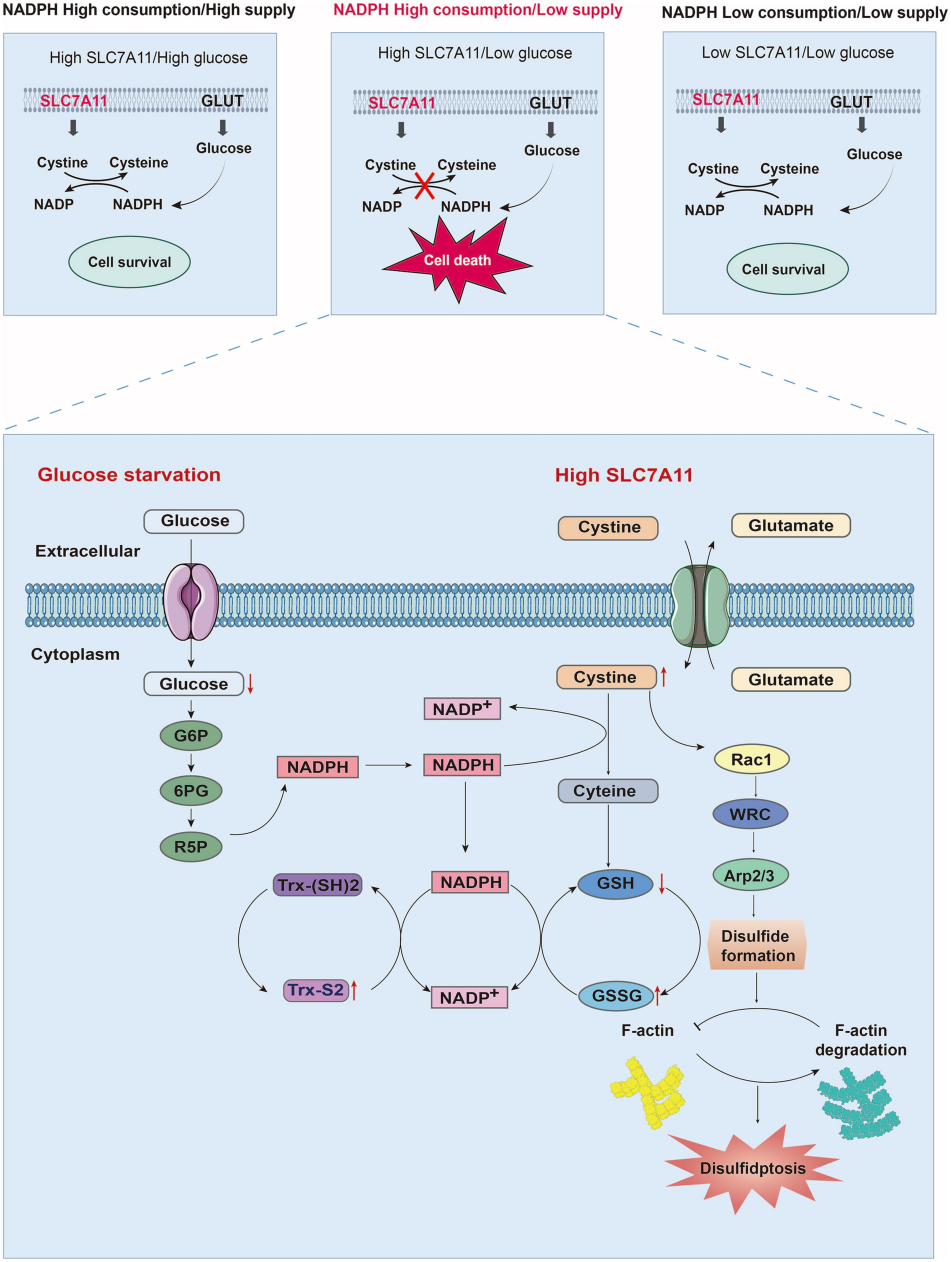
Mechanisms of disulfidptosis. By analyzing the relationships among the expression level of SL7A11, NADPH metabolism, and glucose levels, the factors necessary for cell death were explored. When SLC7A11 and glucose are overexpressed or decreased simultaneously, cell survival occurs. However, only when high SLC7A11, low glucose, and NADPH depletion are present simultaneously can they lead to cystine/cysteine conversion disorders, the accumulation of disulfides, and ultimately disulfidptosis. When glucose starvation blocks the production of NADPH through the pentose phosphate pathway, a large amount of intracellular cysteine input through high expression of SLC7A11 depletes NADPH. Owing to the insolubility of cystine, NADPH is needed as a reducing force to decompose it into cysteine. The low supply of NADPH also blocks the process of cystine conversion to cysteine, leading to a large accumulation of cystine and activating the Rac1-WRC-Arp2/3 pathway. Abnormal disulfide bonds are formed in actin cytoskeleton proteins, and F-actin is broken down, ultimately leading to disulfidptosis. Moreover, depletion of NADPH can also hinder GSH/GSSG conversion and Trx-(SH) 2/Trx-S2 conversion, ultimately leading to an imbalance in the intracellular glutathione and thioredoxin antioxidant systems. Abbreviations: GLUT: glucose transporter; Trx-(SH) 2: thioredoxin reduced; Trx-S2 thioredoxin oxidized; NADP+: nicotinamide adenine dinucleotide phosphate, reduced form; NADPH: nicotinamide adenine dinucleotide phosphate; G6P: glucose-6-phosphate dehydrogenase; 6PG: 6-phosphogluconate; R5P: ribose-5-phosphate dehydrogenase; GSH: glutathione; GSSG: glutathione oxidized.

Trx and Grx systems are widely distributed in the brain and participate in the formation, transfer, and isomerization of disulfide bonds through thiol-disulfide exchange reactions (Sousa et al., 2019; Aon-Bertolino et al., 2011). Trx and GSH/Grx systems are distributed in the cortex, striatum, hippocampus, etc., and play crucial roles in the cellular defense against oxidative stress in hypoxia-induced ischemic injury. Additionally, redox reactions are regulated by the oxidoreductases Trx and Grx, which are specific targets of signal transduction pathways associated with the stress response (Jiménez et al., 2024). Moreover, Trx and Grx preserve the reduced state of the cell by reducing oxidized thionine residues in actin to protect the morphology of the cytoskeleton (Meyer et al., 2012). Consequently, the Trx and Grx systems maintain the balance of thiol-disulfide interactions in the reduction/oxidation conversion of protein forms, which act as target signaling pathways to regulate disulfide stress and may act as crucial triggers to suppress disulfidptosis.

## 3 Disulfidptosis-related programmed cell death

There are currently many types of cell death, such as pyroptosis, apoptosis, and autophagy, but only ferroptosis and cuproptosis are related to disulfidptosis. The cell death in three types of cell death, namely disulfidptosis ferroptosis, and cuproptosis, is related to redox homeostasis. One or more key factors may act as switches for cells to oscillate between the three modes of cell death. It is speculated that this common switch is cystine to cysteine (Wang et al., 2024). Recent research has found that a new type of carrier free nanoparticle has been developed to effectively treat cancer through the combined effect of ferroptosis and cuproptosis (Zhu et al., 2024a).

### 3.1 Ferroptosis

The DIXON group first postulated that ferroptosis, a type of programmed cell death initiated by lipid peroxidation, depends on iron (Seibt et al., 2019). Some proteins implicated in disulfidptosis are also associated with the initiation and progression of ferroptosis. First, System Xc (cystine/glutamate antiporter) is constructed from the heavy chain SLC3A2 and light chain SLC7A11 (xCT) (Tu et al., 2021). SLC7A11, following its overexpression, imports cystine to suppress ferroptosis through GSH biosynthesis and antioxidant defense (Koppula et al., 2021). Moreover, the upregulation of SLC7A11 contributes to the emergence of disulfidptosis by increasing the input of cystine and causing an imbalance in the cystine/cysteine ratio, which in turn promotes disulfidptosis (Liu X. et al., 2024). Second, NADPH is required for inhibiting lipid oxidation and serves as an indicator of ferroptosis in various types of tumor cells (Wang et al., 2023). NADPH depletion also induces a high cystine/cysteine ratio, leading to disulfide stress, which facilitates disulfidptosis (Dixon et al., 2012). Another relevant mechanism includes the disulfide-glutathione redox couple (GSH/GSSG) (Ursini and Maiorino, 2020). GSH inhibits cellular ferroptosis by stimulating the production of glutathione peroxidase (GPX4) to reduce lipid peroxidation products (Dixon et al., 2012). Additionally, GSH and GSSG act as crucial constituents of the thiol/disulfide redox system (Circu and Aw, 2011), which may be closely related to disulfide stress. During ferroptosis, NADPH is a key regulator that acts as a cofactor and functions along with GRX to reverse the conversion of GSSG to GSH, sustain the balance between GSH and GSSG, and inhibit lipid peroxidation damage (Seibt et al., 2019; Lu, 2013). During signal cascades, enzymes in the Trx family (Trxs and Grxs) also play a significant role in maintaining the balance of oxidation–reduction reactions, such as the ratio of GSH/GSSG, by regulating thiol/disulfide conversion (Aoyama, 2021; Musaogullari and Chai, 2020). Consequently, the regulation of the expression of the membrane transporter SLC7A11, NADPH depletion because of glucose starvation, and subsequent disruption of the balance of thiol/disulfide bonds may serve as a common link between disulfidptosis and ferroptosis (Figure 2), potentially involving intersecting molecular pathways.

### 3.2 Cuproptosis

Cuproptosis is a newly recognized type of cell death, and the process involves reliance on copper, accumulation of proteins modified with fatty acids, and reduction of Fe-S cluster proteins (Chen et al., 2022). During cuproptosis, copper accumulation may be regulated by ferredoxin 1 (FDX1) and lipoic acid synthase (LIAS) (Tsvetkov et al., 2022). FDX1 interferes with redox homeostasis to induce cuproptosis in endometriosis, which is mediated by glucose-6-phosphate (G6PD); these changes suppress the proliferation and metastasis of endometrial cells (Lu et al., 2023). Moreover, a previous study revealed that G6PD significantly affects REDOX homeostasis by regulating glycolytic flux through the pentose phosphate pathway (PPP) (Garcia et al., 2021). It also produces NADPH, which is an essential cofactor for glutathione (GSH/GSSG) conversion (Luzzatto et al., 2020). Some related studies have reported that p53 regulates the metabolism of glycolysis and oxidative phosphorylation by acting as the rate-limiting enzyme of the PPP by binding to glucose-6-phosphate dehydrogenase (G6PD) (Vousden and Ryan, 2009). Furthermore, p53 suppresses NADPH production by inhibiting malic enzyme or G6PD (Jiang et al., 2011). Consequently, ferroptosis, cuproptosis, and disulfidptosis may involve interrelated crosstalk mechanisms involving glycolysis and oxidative phosphorylation in the context of circulatory disturbance.

## 4 Research on disulfidptosis-associated CNS disease

### 4.1 Neurodegenerative disease

#### 4.1.1 Alzheimer’s disease

Alzheimer’s disease (AD) is a prevalent condition characterized by progressive degeneration of the brain, which may involve an increase in oxidative stress, a decrease in antioxidant enzymes, and consequent disruption of the redox dynamic balance (Yu et al., 2024). Eight disulfidptosis-related genes (DRGs) significantly affect the beginning and progression of AD. Specifically, the activity of the SLC7A11, SLC3A2, and GYS1 genes increases, whereas the activity of the OXSM, NUBPL, NDUFA11, NCKAP1, and LRPPRC genes decreases (Zhu et al., 2023) (Table 1). Additionally, a differential analysis of the gene expression matrix of AD revealed seven characteristic genes associated with the breakage of disulfide bonds, including MYH9, IQGAP1, ACTN4, DSTN, ACTB, MYL6, and GYS1, which accurately assess subtypes of AD and diagnose AD (Ma et al., 2023). Some studies have suggested that dynamic disruption of the actin cytoskeleton occurs during the progression of AD. A detailed understanding of the mechanisms of the actin cytoskeleton can pave the way for developing innovative synapse-targeted therapeutic interventions and identifying new biomarkers to monitor synaptic loss in Alzheimer’s disease (Pelucchi et al., 2020). Thioredoxin-1 (Trx-1) is a multifunctional molecule that has anti-inflammatory properties in human tissues and plays significant neuroprotective roles in AD (Jia et al., 2024). In addition, the dysregulation of the thioredoxin system increases susceptibility to cell death, and changes in Trx and TrxR levels are significantly associated with the progression of AD (Qaiser et al., 2024).

**Table 1:** Disulfidptosis-related genes in the CNS.

Disease	Disulfidptosis-related genes
Neurogliomas	SLC3A2, NDUFA11, 0XSM, NUBP1, LRPPRC, RPN1, GYSI
Alzheimer’s disease	SLC7A11, SLC3A2, GYSI, OXSM, NUBPL, NDUFA11, NCKAP1, LRPPRC
Ischemia stroke	SLC2A3, SLC2A14, SLC7A11, NCKAP1

Research on neurodegenerative, neuroinflammatory, and neuro-oxidative stress in related brain diseases revealed that genetic and environmental factors affect Trx function and that the Trx system may be an important target for disease intervention and treatment (Bjørklund et al., 2022). Glutaredoxin (Grx) 1 regulates redox signal transduction and protein redox homeostasis by catalyzing reversible s-glutathione modification, thereby exerting neuronal effects (Gorelenkova and Mieyal, 2019). Therefore, controlling oxidative stress responses and maintaining redox balance through the Trx and Grx systems are highly important for the prevention and treatment of Alzheimer’s disease, which prompted us to further explore the correlation between disulfide cell death and these processes.

#### 4.1.2 Parkinson’s disease

Parkinson’s disease is also a prevalent neurodegenerative disease characterized by a progressive decrease in neurological function (Bloem et al., 2021). A previous study revealed that significant alterations in the distribution of antioxidant enzymes and high levels of free radicals play important roles in the development of Parkinson’s disease (Li et al., 2022). The accumulation of ROS leads to nigrostriatal neuronal death in PD (Trist et al., 2019). Additionally, dynamic thiol-disulfide homeostasis is disrupted in patients with Parkinson’s disease (Vural et al., 2017). The expression of the Parkinson’s disease-related gene Parkin contributes to the network of sulfhydryl groups available in the cell, including glutathionylation, which involves reversible post-translational modifications of selected cysteine residues (El et al., 2023). Moreover, a cluster analysis of S-glutathionylated targets, which are known to play a role in apoptosis and inflammation according to the Gene Ontology (GO) collection or Kyoto Encyclopedia of Genes and Genomes (KEGG), revealed a putative overlapping association between Grx1 and neurodegenerative diseases (Gorelenkova and Mieyal, 2019). Higher amounts of TRX and GRX were found to protect cells from the reactive dopamine metabolite 6-OHDA-benzenediol, which is critical for neuronal survival in dopamine-induced cell death (Arodin et al., 2014). Bioinformatics analysis and *in vitro* and *in vivo* experiments have shown that the abnormal balance of thiol/disulfide bonds leading to oxidative stress is the pathological mechanism underlying neuronal damage in neurodegenerative diseases. Therefore, further investigation of its association with disulfidptosis is highly important.

### 4.2 Neurogliomas

Neurogliomas are the predominant malignant tumors that originate in the CNS (Ostrom et al., 2023). Recently, the disulfidptosis related genes (DRGs) SLC3A2, NDUFA11, OXSM, NUBPL, LRPPRC, RPN1, and GYS1 were found to be significantly associated with glioma cells. The upregulation of the disulfidptosis-related gene SLC3A2 influences immune cell infiltration in gliomas, notably macrophage infiltration, and impacts tumor migration and invasion, consequently affecting the tumor microenvironment (Xu Y. et al., 2024). Additionally, a study revealed that high expression of LRPPRC was positively correlated with a favorable prognosis for gliomas, but increased expression of RPN1 and GYS1 was associated with an unfavorable prognosis (Guo et al., 2023). Additionally, thioredoxin NADPH reductase (TrxR) plays a crucial role in the progression of malignancies (Branco et al., 2020). The redox function of thioredoxin is largely dependent on TrxR, with NADPH serving as an electron donor (Saccoccia et al., 2014). SLC7A11 was identified as the gene that is most significantly associated with disulfidptosis in tumors (Huang et al., 2023; Xu B. et al., 2024). SLC7A11 expression in gliomas plays a significant role in tumorigenesis, tumor progression, and resistance to conventional chemotherapy. Several studies have shown that glioma cells upregulate the expression of SLC7A11, which regulates glutathione production and glioma growth (Polewski et al., 2016). Nrf2 is a transcription factor that is sensitive to redox changes and capable of regulating the expression of the intracellular redox balance proteins GPX4 and SLC7A11 in glioma cells (Gao et al., 2020). Chrysomycin A (Chr-A) further altered the levels of nicotinamide adenine dinucleotide phosphate (NADPH), leading to oxidative stress and downregulation of Nrf-2 to inhibit glioblastoma (Liu D. N. et al., 2024). The upregulation of disulfidptosis related genes SLC3A2 and SLC7A11 promotes the occurrence and progression of glioma tumors, therefore, a signal target of disulfidptosis may be used in the therapeutic schedule for neurogliomas.

### 4.3 Ischemic stroke

#### 4.3.1 Factors associated with disulfidptosis in stroke patients: glucose starvation and SLC7A11

Stroke includes both ischemic and hemorrhagic stroke; both lead to abnormal cerebral blood flow and disrupt the delivery of oxygen and glucose, resulting in cellular dysfunction, such as mitochondrial oxidative phosphorylation and bioenergetic stress (An et al., 2021; Zheng J. et al., 2018). In ischemic stroke, blood vessel blockage results in glucose and oxygen deficiency, which triggers several biological response pathways and ultimately leads to irreversible brain damage (Sharma et al., 2022). Trx/TrxR is an NADPH-dependent cellular thiol reduction-antioxidant system, and the PPP and glucose oxidative decomposition are the main sources of NADPH (Wang et al., 2022). Reduced coenzyme II (NADPH) is the reduced form of NADP+ and is derived mainly from the pentose phosphate pathway (PPP) of glucose oxidative degradation, and NADPH depletion is also associated with ischemic stroke (Zhang and Peng, 2014). Clinical studies have proposed that changes in TyG-BMI, calculated via the formula [triglyceride (mg/dL) × fasting blood glucose (mg/dL)/2] BMI (kg/m2), are associated with the prognosis of stroke (Huo et al., 2023; Yang et al., 2023). Thus, glucose starvation, which is an initial factor necessary for disulfidptosis, occurs in cerebral ischemia. Recently, differential gene expression analysis revealed that four DRGs (SLC2A3, SLC2A14, SLC7A11, and NCKAP1) are associated with stroke (Liu S. P. et al., 2024). Single cell analysis shows that the seven types of DRGs (ACTB, IQGAP1, FLNA, PDLIM1, MYH10, INF2 and SLC7A11) are mainly distributed in the immune cell types of ischemic stroke (Qin et al., 2023). Recent studies have found that SLC7A11 expression inhibits M1 polarization in microglia while promoting M2 polarization in OGD models (Zhao et al., 2024). A study revealed that increased SLC7A11 expression helps enhance GSH synthesis, which increases resistance to oxidative damage and prevents neuronal ferroptosis during cerebral ischemia (Li et al., 2023; Yuan et al., 2021). Therefore, SLC7A11, a subunit of the Xc system that imports cystine through the export of glutamate at a 1:1 ratio, may be involved in the crosstalk between ferroptosis and disulfidptosis in ischemic stroke. Studies have shown the protective effects of Peroxiredoxin 1 (PRDX1) on stroke through disulfidptosis and the ischemic postconditioning’s (IPostC) mechanism. This marks an important step forward in stroke research and potential treatment development (Liu S. et al., 2024). A precursor of cysteine, known as L-2-oxothiazolidine-4-carboxylic acid (OTC), decreases neurobehavioral performance in stroke models (Liu Y. et al., 2020). Empirical clinical data have demonstrated that individuals exhibit considerably increased serum concentrations of homocysteine and cysteine in the acute phase of atherothrombotic stroke (Salemi et al., 2009)^.^ A study reported that cysteine levels can increase 10–13-fold over 8 h in the ischemic hippocampus and striatum, which are derived largely from GSH (Slivka and Cohen, 1993). The accumulation of cysteine, which originates from GSH, substantially increased excitotoxic damage and disrupted the balance of GSH/GSSG tissue damage caused by stroke in a rat model (Qu et al., 2006).

#### 4.3.2 Disulfidptosis-associated redox state: thiol/disulfide bond regulation in stroke

Disulfidptosis is a method of cell death that involves a reduction–oxidation (redox) reaction and the production of disulfide bonds (Wang et al., 2023). Moreover, preliminary research has indicated that alterations in thiol levels during oxidative stress may be associated with the size of the infarct resulting from ischemic stroke. Additionally, reversing thiol deficiency and restoring the thiol-disulfide balance that reduce disulfide accumulation and inhibit oxidative damage, which can impede the damage caused by ischemic stroke (Bektas et al., 2016). Trx/TrxR is an NADPH-dependent cellular thiol reduction antioxidant system associated with disulfide stress. NADPH originates from the PPP, in which the oxidative decomposition of glucose is affected by hypoxia (Wang et al., 2022). The Trx system is crucial for regulating apoptosis, redox status, and antioxidant defenses (Matsuzawa, 2017). Therefore, Trx may have a neuroprotective function in individuals suffering from acute ischemic stroke, and serum Trx levels are novel diagnostic and prognostic indicators that are also closely related to the severity of intracranial hemorrhage and long-term mortality. Additionally, the overexpression of Grx1 decreases the level of S-glutathionylation, reduces the depletion of GSH and the formation of disulfide bonds, and suppresses neuron damage during focal ischemia (Takagi et al., 1999).

#### 4.3.3 Disulfidptosis-associated signaling pathway: Nrf2/Trx regulation in stroke

Nuclear factor E2-associated factor 2 (Nrf2) is a transcription factor that binds to Kelch-like ECH-associated protein 1 (KEAP1) (Suzuki et al., 2023). In the nucleus, they function along with other coactivators to initiate the transcription of target genes under certain stimuli, such as oxidative stress, disrupting binding (Baird and Yamamoto, 2020). The Keap1-Nrf2 system is a sulfhydryl-based sensor-effect device that maintains redox homeostasis and is vital to cellular defense against exogenous and endogenous oxidative and electrophilic stress (Yamamoto et al., 2018). Endogenous Nrf2 is activated after ischemic stroke, resulting in an initial increase in the expression of overall Nrf2 protein and a subsequent decrease in the ischemic zone (Tian et al., 2020; Yang et al., 2009). Oxygen–glucose deprivation/reperfusion (OGD/R)-induced ferroptosis can be reversed by accelerating the transcription of GPX4 via the Nrf2-SLC7A11 signaling pathway (Liu et al., 2023b), which may be associated with disulfidptosis. After transient middle cerebral artery occlusion in Nrf2 gene knockout mice, the induction and activation of antioxidant enzymes are inhibited, resulting in a larger stroke area (Zhang et al., 2017). A study revealed that Nrf2 siRNA injection decreases the protein and mRNA expression of Trx1 in middle cerebral artery occlusion (MCAO) model rats; therefore, Trx1 is regulated by Nrf2, which has a neuroprotective function (Li et al., 2015). Under ischemic conditions, Nrf2 can regulate the disulfidptosis-related proteins SLC7A11 and the Trx system. Therefore, whether Nrf2 is a regulatory target of disulfidptosis after cerebral ischemia deserves further investigation. Further investigations are needed to determine which of these factors are important target signals of disulfidptosis and whether a temporal sequential relationship exists between the occurrence of disulfidptosis and ferroptosis in stroke. These studies suggest that an imbalance in thiol/disulfide redox reactions during disulfidptosis might constitute a novel pathogenic pathway of stroke or other disorders of the CNS; deeper insights into the mechanism underlying disulfidptosis may provide new targets and new treatments for CNS diseases.

## 5 Implications of disulfidptosis treatment

Research on the pathogenesis and treatment of disulfidptosis is still in the early stage and has focused mainly on various types of cancer. A review of the literature on drug intervention for disulfidptosis target genes and signaling pathways may provide new directions for research on CNS therapy. N-acetylcysteine (NAC) have antioxidant, anti-inflammatory, and neuroprotective processes in the CNS (Santos et al., 2017). One study suggested that NAC acts as an antioxidant to increase the intracellular concentration of GSH, which is the critical biological thiol responsible for maintaining the cellular redox balance (Tenório et al., 2021). NAC promotes the regeneration of free thiols through disulfide exchange, preventing the accumulation of cystine or other disulfides under glucose starvation (e.g., Cys-Cys + NAC → Cys + NAC-Cys) (Sarıtaş et al., 2024). In recent clinical trials on acute ischemic stroke, it has been found that intravenous injection of N-acetylcysteine can enhance the safety and efficacy of recombinant tissue plasminogen activator (rtPA or alteplase) adjuvant therapy (Zhao et al., 2017). In summary, in the carrier experiment, NAC can increase GSH and reduce the accumulation of cystine and disulfide. We believe that NAC may have a therapeutic effect on inhibiting neuronal disulfidptosis after CNSD. Lipoic acid (α-LA), also known as thioctic acid, includes a reduced (dihydro-lipoic acid, DHLA) form, which exerts its neuroprotective effect on neurodegenerative disorders by inhibiting the formation of oxidizing material and promoting neurotransmitters (Santos et al., 2017). Liraglutide (LIRA), a glucagon-like peptide-1 (GLP-1) analog widely used in clinical applications, enhances the expression of Trx, Nrf2, and p-Erk1/2 to establish a protective effect against neurodegenerative diseases (Liu J. et al., 2020). Salidroside (Sald), a traditional Chinese medicine, may suppress oxidative stress by inducing Trx and peroxiredoxin-I (PrxI) in neuroblastoma (Zhang et al., 2010). Ebselen, a synthetic organoselenium compound, protects neurons from damage caused by free radicals by interacting with thiols, peroxynitrites, and hydroperoxides (Wang J. et al., 2020). The antioxidant Dl-3-n-butylphthalide can hinder the NLRP3 inflammasome and decrease the severity of AD-like symptoms by affecting the Nrf2-TXNIP-TrX pathway (Wang et al., 2019). Disulfidptosis may be an important pathological mechanism in neurodegenerative diseases. In response to the imbalance of intracellular sulfur metabolism, thiol and disulfide balance, as well as the systemic regulation of Trx and Grx and the cross-talk between cell death after the occurrence of the disease, effective interventions and treatment drugs have been explored to identify pathways and multiple approaches for treating neurodegenerative diseases. Disulfidptosis is a novel mode of programmed cell death, and the investigation and intervention of pathological mechanisms may reveal various effective targets. This information can be used to treat associated CNS diseases effectively (Table 2).

**Table 2:** Disulfidptosis-related therapeutic drugs.

Medicine	Source	Mechanism of action	Corresponding disease
NAC	Thermal compound	Increase concentration of GSH	CNS
Lipoic acid	Organic compounds	Inhibits the formation of oxides and promotes neurotransmitters	Neuroelegenerative disease
LIRA	Synthetic acylated GIP-1 analogs	Enhanced expression of Trx, Nrf2, P-Erk1/2	Neuroelegenerative disease
Salidroside	Botany	Trx Prx-1	Neuroblastoma
Ebselen	Organic chemicals	Thiols, peroxynitries hydroperoxides	Neuroelegenrative disease
Dl-3-n-butylphthalide	Apium graveolens Linn	NLRP3	Alzheimer’s disease

## 6 Outlook and conclusions

In this review, we emphasized that NADPH metabolism, high SLC7A11 expression, and the cystine/cysteine balance are important metabolic features of disulfidptosis. By describing the endogenous and exogenous elements involved in novel cell death disulfidptosis and identifying associations between disulfidptosis and CNS disorders, such as neurodegenerative diseases, gliomas, and ischemic stroke, this review summarizes disulfidptosis-related genes involved in neurodegenerative diseases, gliomas, and ischemic strokes and discusses how disulfide stress and redox imbalance contribute to the onset and progression of CNSD. The Trx and Grx systems, which play antioxidant defense roles during disulfide stress, are involved in neurological diseases. In the future, we can use bioinformatics analysis and *in vitro* and *in vivo* experiments to detect target genes and protein proteins associated with disulfidptosis in neurological diseases to search for clinical biomarkers for treatment. We suggest that disulfidptosis might be a crucial novel pathological mechanism underlying CNS diseases. Moreover, some similarities between disulfidptosis and ferroptosis/cuproptosis may reveal new insights into the pathogenesis of CNS diseases and help develop more comprehensive therapeutic strategies. Therefore, it is necessary to conduct a thorough analysis of other pathways involved in mediating disulfidptosis.

However, the potential mechanism of disulfidptosis still needs further exploration, and this innovative approach will provide a basis to overcome future challenges. Will disulfidptosis only occur in the actin cytoskeleton, and are other proteins sensitive to disulfide stress? What are the differences between disulfide bonds and other protein post-translational modifications, especially with regard to glutathionylation? In future basic and clinical research, can we select appropriate therapeutic drugs for patients with central nervous system diseases based on their susceptibility to disulfidptosis? Exploring the mechanism underlying disulfidptosis and its contribution to various central nervous system diseases has crucial theoretical and practical value for identifying effective treatment strategies.
